# Supplementary material for: Drivers of the opioid crisis: An appraisal of financial conflicts of interest in clinical practice guideline panels at the peak of opioid prescribing
Source: PLoS One. 2020 Jan 24;15(1):e0227045. doi: 10.1371/journal.pone.0227045 (PMC6980493; doi:10.1371/journal.pone.0227045)
Supplement: S1 Table — Table with information on sponsor/author, guidelines name, references, elements known to introduce bias, number of red flags and uncertain rating and notes for each guideline. (DOCX) [file pone.0227045.s001.docx]

**S1 Table. Master Data Extraction Sheet. Table with information on sponsor/author, guidelines name, references, elements known to introduce bias, number of red flags and uncertain rating and notes for each guideline.**

| Sponsor/Author | Guideline | Ref | Sources | Element known to introduce potential bias | Red flags | Notes |
| --- | --- | --- | --- | --- | --- | --- |
| American College of Occupational and Environmental Medicine (ACOEM) | ACOEM Guidelines for Chronic Use of Opioids (2011) | [1] | Email communication  Supplementary materials [2] | Sponsor is conflicted:  Industry/unknown sponsor:  Committee chair conflicted  Multiple panelists conflicted  Committee stacking  Limited methodologist  No external review  No non-physicians or patients | No flag  N/A  No flag  No flag  Red flag  No flag  No flag  Red flag | Not stated. No pharma industry sponsors in searches  N/A  Not stated: ACOEM provided names and COI via email. No pharma industry conflicts reported for chairs or found in searches  Not stated: ACOEM provided names and COI via email. No pharma industry conflicts reported committee members  15 panel members, 1 addictions expertise (1/15 =7%)  Standing methodology committee that provides guidance/oversight.  Sent for external review according to supplementary materials: ACEOM guidelines methodology document  Two non-physicians, no patients |
| American Geriatrics Society (AGS) | Pharmacological Management of Persistent Pain in Older Persons and Management of Persistent Pain in Older Persons (2009) | [3,4] |  | Sponsor is conflicted:  Industry/unknown sponsor:  Committee chair conflicted  Multiple panelists conflicted  Committee stacking  Limited methodologist  No external review  No non-physicians or patients | Red Flag  N/A  Red Flag  Red flag  No flag  Red flag  No flag  Red flag | Not stated. Has pharma industry sponsors in searches  N/A  Stated: none but COI for chair with Purdue and Endo pharma companies (2007) found in searches  Stated: many members with COI with pharma  10 members, 1 with expertise in addictions (10%)  No methodologist in lead role or providing oversight  Sent for external review  Two non-physicians, no patients |
| American Pain Society and American Academy of Pain Medicine (APS and AAPM) | Clinical Guidelines for the Use of Chronic Opioid Therapy in Chronic Noncancer Pain (2009) | [5–7] |  | Sponsor is conflicted:  Industry/unknown sponsor:  Committee chair conflicted  Multiple panelists conflicted  Committee stacking  Limited methodologist  No external review  No non-physicians or patients | Red Flag  N/A  Red Flag  Red Flag  Red flag  No flag  No flag  Red flag | Not stated. Pharma industry sponsors reported in annual report  N/A  Stated: Co-chairs- one with conflicts  Stated: 7 with pharma industry conflicts  14 members, 1 addiction MD (1/14) (7%)  Chair is methodologist, expert in systematic reviews  Sent for external review  Three non-physicians, no patients |
| American Society of Anesthesiologists (ASA) | Practice Guidelines for Chronic Pain Management: An Updated Report by the American Society of Anesthesiologists Task Force on Chronic Pain Management and the American Society of Regional Anesthesia and Pain Medicine (2010) | [8] | Email communication | Sponsor is conflicted:  Industry/unknown sponsor:  Committee chair conflicted  Multiple panelists conflicted  Committee stacking  Limited methodologist  No external review  No non-physicians or patients | Red flag  N/A  No flag  Uncertain*  Red flag  No flag  Red flag  Red flag | Not stated. Has pharma industry sponsors in searches  N/A  None stated, no pharma industry conflicts in searches  Not stated: in guideline materials, when emailed, they refused to provide and put me in contract with their lawyer  13 members, none with addictions expertise  Two consulting methodologist from ASA standards committee  Review only within the ASA/ASRA and their national meetings  No non-physicians or patients on committee |
| American Society of Interventional Pain Physicians (ASIPP) | American Society of Interventional Pain Physicians (ASIPP) Guidelines for Responsible Opioid Prescribing in Chronic Non-Cancer Pain (2012) | [9,10] |  | Sponsor is conflicted:  Industry/unknown sponsor:  Committee chair conflicted  Multiple panelists conflicted  Committee stacking  Limited methodologist  No external review  No non-physicians or patients | No flag  N/A  No flag  No flag  Red flag  Red flag  No flag  Red flag | Not stated. No pharma funding found in searches  N/A  None stated, no pharma industry conflicts in searches  Stated: 1 author had conflicts of interest with pharma industry (1/55)  55 members, no addiction experts  No methodologist in lead role/oversight role  Sent for external review  Some non-physicians, no patients |
| National Opioid Use Guideline Group (NOUGG) | Canadian Guideline for Safe and Effective Use of Opioids for Chronic Non-Cancer Pain (2010) | [11–14] |  | Sponsor is conflicted:  Industry/unknown sponsor:  Committee chair conflicted  Multiple panelists  Committee stacking  Limited methodologist  No external review  No non-physicians or patients | No flag  N/A  Red flag  Red flag  No flag  No flag  No flag  Red flag | Stated funded by governmental grant to NOUGG, a subcommittee of Federation of Medical Regulatory Authorities of Canada  N/A  Stated one had conflicts of interest with pharma (two immediate family members worked for pharma industry)  Stated: 3/5 conflicts of interest with pharma industry  5 members, 3/5 addictions expertise  Chair is epidemiologist  Sent for external review  No patients on committee, some non-physicians |
| Colorado Division of Workers’ Compensation (Colorado DWC) | Chronic Pain Disorder Medical Treatment Guidelines (2011) | [15] | Email communication | Sponsor is conflicted:  Industry/unknown sponsor:  Committee chair conflicted  Multiple panelists conflicted  Committee stacking  Limited methodologist  No external review  No non-physicians or patients | No flag  N/A  Uncertain*  Uncertain*  No flag  Red flag  Red flag  Red flag | Not stated, no pharma industry funding found in searches  N/A  Not Stated: Email response Colorado DWC, chairs not named, COI not collected at time  Not stated: Not done at time according to email response from Colorado DWC  15 members, 2 addiction expertise (2/15) (13%)  None listed  No external review mentioned  Non physicians, no patients |
| Fine et al, 2009 | Establishing “Best Practices” for Opioid Rotation: Conclusions of an Expert Panel (2009) | [16] |  | Sponsor is conflicted:  Industry/unknown sponsor:  Committee chair conflicted  Multiple panelists conflicted  Committee stacking  Limited methodologist  No external review  No non-physicians or patients | N/A  Red flag  Red flag  Uncertain*  Uncertain*  Red flag  Red flag  Red flag | N/A  Stated: Supported by an unrestricted grant from Endo Pharmaceuticals  Stated: authors state no conflicts, searches show both had conflicts with pharma industry in 2008 including Purdue  Stated: “The authors declare no financial conflicts with respect to this work”.  12 members, no addiction experts  Both chairs are content experts  No external review mentioned  Two non-physicians, no patients |
| Institute for Clinical Systems Improvement (ICSI) | Assessment and Management of Chronic Pain (2011)  **via email because no longer available online | [17] |  | Sponsor is conflicted:  Industry/unknown sponsor:  Committee chair conflicted  Multiple panelists conflicted  Committee stacking  Limited methodologist  No external review  No non-physicians or patients | No flag  N/A  No flag  No flag  Red flag  Red flag  No flag  No flag | Not stated but no private sponsorship, as per its own guidelines. No pharma funding found in searches  N/A  Stated: none and none in searches  Stated: 1 with pharma conflict- stock holdings and speakers bureau  10 members, no addiction experts  Co-leads are content experts, ICSI staff do literature search/review  Sent for external review  Have patient advisory group and non-physician members |
| University of Michigan Health System (UMHS) | Managing Chronic Non-Terminal Pain in Adults, Including Prescribing Controlled Substances (2012)  ** via email because no longer available online | [18] |  | Sponsor is conflicted:  Industry/unknown sponsor:  Committee chair conflicted  Multiple panelists conflicted  Committee stacking  Limited methodologist  No external review  No non-physicians or patients | No flag  N/A  No Flag  No Flag  Red flag  Red flag  Red flag  Red flag | Not stated, no pharma industry funding found in searches  N/A  Stated no conflicts and none in searches  Stated: 1 with conflicts  9 members, no addiction experts  Leads are content experts, used literature review from VA in 1998/2002  Not stated  Non-physicians, no patients |
| Utah Department of Health (UDOH) | Utah Clinical Guidelines on Prescribing Opioids for Treatment of Pain (2009) | [19,20] |  | Sponsor is conflicted:  Industry/unknown sponsor:  Committee chair conflicted  Multiple panelists conflicted  Committee stacking  Limited methodologist  No external review  No non-physicians or patients | No flag  N/A  No flag  No flag  No flag  No flag  No flag  Red flag | Not stated. None found in searches  N/A  Stated: no conflicts stated, and none found with searches  Stated: one panelist has many conflicts with pharma  12 members, 2/12 are addiction experts (17%)  Chair is methodologist  Sent for external review  Five non-physicians, no patients |
| Veterans Affairs and Department of Defense (VA/ DoD) | Clinical Practice Guideline for Management of Opioid Therapy for Chronic Pain (2010) | [21] | Email communication  Supplementary materials [22] | Sponsor is conflicted:  Industry/unknown sponsor:  Committee chair conflicted  Multiple panelists conflicted  Committee stacking  Limited methodologist  No external review  No non-physicians or patients | Red flag  N/A  Red flag  Uncertain*  No flag  No flag  No flag  Red flag | Not stated. Has pharma industry sponsors found in searches  N/A  Not stated. Searches: one chair on national board directors 2009 for APF (90% pharma-funded including Purdue)  Not stated, email response- no relevant conflicts but no additional information provided  20 members, 2 addiction specialists (2/20) (10%)  Have oversight methodology committee  Sent for external review according to supplementary material  Two non-physicians, no patients |
| Work Loss Data Institute (WLDI) | Pain (Chronic) (2011) | [23] | Email communication | Sponsor is conflicted:  Industry/unknown sponsor:  Committee chair conflicted  Multiple panelists conflicted  Committee stacking  Limited methodologist  No external review  No non-physicians or patients | No flag  N/A  No flag  No flag  Red flag  Red flag  No flag  Red flag | Not stated. No pharma industry sponsors in searches  N/A  Not stated: From email: no conflicts. Nothing in searches  Not stated: Emailed me list of participants, no pharma COI  17 members, no addiction experts  No lead or oversight by methodologist  Yes, sent for external review  Non physicians, but no patients |
|  |  |  |  |  |  |  |

APF = American Pain Foundation, ASRA= American Society of Regional Anesthesia and Pain Medicine, COI = conflict of interest, N/A = not applicable, pharma = pharmaceutical company

* Element that could not be confidently appraised with available information

**References**

1. American Coll of Occupational and Environmental Medicine. ACOEM Guidelines for Chronic Use of Opioids. American Coll of Occupational and Environmental Medicine; 2011. Available: https://www.nhms.org/sites/default/files/Pdfs/ACOEM%202011-Chronic%20Pain%20Opioid%20.pdf

2. American College of Occupational and Environmental Medicine. Methodology for ACOEMʼs Occupational Medicine Practice Guidelines, 2011 revision. 2011. Available: https://www.acoem.org/gmc.aspx

3. American Geriatrics Society Panel on Pharmacological Management of Persistent Pain in Older Persons. Pharmacological management of persistent pain in older persons. J Am Geriatr Soc. 2009;57: 1331–1346. doi:10.1111/j.1532-5415.2009.02376.x

4. AGS Panel on Persistent Pain in Older Persons. The management of persistent pain in older persons. J Am Geriatr Soc. 2002;50: S205-224.

5. Chou R. 2009 Clinical Guidelines from the American Pain Society and the American Academy of Pain Medicine on the use of chronic opioid therapy in chronic noncancer pain: what are the key messages for clinical practice? Pol Arch Med Wewn. 2009;119: 469–477.

6. Chou R, Fanciullo GJ, Fine PG, Adler JA, Ballantyne JC, Davies P, et al. Clinical Guidelines for the Use of Chronic Opioid Therapy in Chronic Noncancer Pain. The Journal of Pain. 2009;10: 113-130.e22. doi:10.1016/j.jpain.2008.10.008

7. American Pain Society, American Academy of Pain Medicine. Guideline for the Use of Chronic Opioid Therapy in Chronic Noncancer Pain: Evidence Review. American Pain Society, American Academy of Pain Medicine; 2009. Available: http://americanpainsociety.org/uploads/education/guidelines/chronic-opioid-therapy-cncp.pdf

8. American Society of Anesthesiologists Task Force on Chronic Pain Management, American Society of Regional Anesthesia and Pain Medicine. Practice guidelines for chronic pain management: an updated report by the American Society of Anesthesiologists Task Force on Chronic Pain Management and the American Society of Regional Anesthesia and Pain Medicine. Anesthesiology. 2010;112: 810–833. doi:10.1097/ALN.0b013e3181c43103

9. Manchikanti L, Abdi S, Atluri S, Balog CC, Benyamin RM, Boswell MV, et al. American Society of Interventional Pain Physicians (ASIPP) guidelines for responsible opioid prescribing in chronic non-cancer pain: Part I--evidence assessment. Pain Physician. 2012;15: S1-65.

10. Manchikanti L, Abdi S, Atluri S, Balog CC, Benyamin RM, Boswell MV, et al. American Society of Interventional Pain Physicians (ASIPP) guidelines for responsible opioid prescribing in chronic non-cancer pain: Part 2--guidance. Pain Physician. 2012;15: S67-116.

11. Furlan AD, Reardon R, Weppler C, National Opioid Use Guideline Group. Opioids for chronic noncancer pain: a new Canadian practice guideline. CMAJ. 2010;182: 923–930. doi:10.1503/cmaj.100187

12. National Opioid Use Guideline Group. Canadian Guideline for Safe and Effective Use of Opioids for Chronic Non-Cancer Pain. National Pain Centre; 2010. Available: http://nationalpaincentre.mcmaster.ca/opioid_2010/

13. Kahan M, Mailis-Gagnon A, Wilson L, Srivastava A, National Opioid Use Guideline Group. Canadian guideline for safe and effective use of opioids for chronic noncancer pain: clinical summary for family physicians. Part 1: general population. Can Fam Physician. 2011;57: 1257–1266, e407-418.

14. Kahan M, Wilson L, Mailis-Gagnon A, Srivastava A, National Opioid Use Guideline Group. Canadian guideline for safe and effective use of opioids for chronic noncancer pain: clinical summary for family physicians. Part 2: special populations. Can Fam Physician. 2011;57: 1269–1276, e419-428.

15. Colorado Division of Workers’ Compensation. Chronic Pain Disorder Medical Treatment Guidelines. Colorado Division of Workers’ Compensation; 2011.

16. Fine PG, Portenoy RK, Ad Hoc Expert Panel on Evidence Review and Guidelines for Opioid Rotation. Establishing “best practices” for opioid rotation: conclusions of an expert panel. J Pain Symptom Manage. 2009;38: 418–425. doi:10.1016/j.jpainsymman.2009.06.002

17. Institute for Clinical Systems Improvement. Assessment and Management of Chronic Pain. Bloomington, MN: Institute for Clinical Systems Improvement; 2011. Institute for Clinical Systems Improvement; 2011.

18. University of Michigan. Managing Chronic Non-Terminal Pain in Adults Including Prescribing Controlled Substances. University of Michigan; 2009.

19. Utah Department of Health. Utah Clinical Guidelines on Prescribing Opioids for Treatment of Pain. Utah Department of Health; 2009.

20. Rolfs RT, Johnson E, Williams NJ, Sundwall DN, Utah Department of Health. Utah clinical guidelines on prescribing opioids for treatment of pain. J Pain Palliat Care Pharmacother. 2010;24: 219–235. doi:10.3109/15360288.2010.503265

21. Department of Veterans Affairs, Department of Defense. Clinical Practice Guideline for Management of Opioid Therapy for Chronic Pain. Department of Defense; 2010. Available: http://www.healthquality.va.gov/guidelines/Pain/cot/COT_312_Full-er.pdf

22. Department of Veteran Affairs and Department of Defense. Guidelines for guidelines. 2013.

23. Work Loss Data Institute. Pain (chronic). Work Loss Data Institute; 2011.
