# Supplementary material for: Drivers of the opioid crisis: An appraisal of financial conflicts of interest in clinical practice guideline panels at the peak of opioid prescribing
Source: PLoS One. 2020 Jan 24;15(1):e0227045. doi: 10.1371/journal.pone.0227045 (PMC6980493; doi:10.1371/journal.pone.0227045)
Supplement: Attachment — Submitted filename: PLOS - Opioid Guidelines - July 10, 2019.docx [file pone.0227045.s002.docx]

Thank you for the opportunity to review your manuscript. This manuscript provides an important contribution to the literature, especially as we grow to understand the depths of industry involvement in the current opioid prescribing environment and resultant widespread risks for and real harm. Please find below my line-by-line comments. As a general comment, be sure to refer to *risk* for bias, rather than real bias because real bias was not measured. Accept with minor revisions.

- AS

| Line No./Section | Comment |
| --- | --- |
| Introduction | The population of guidelines is not explicitly stated. The paper refers to both Canadian and US guidelines at line 56, but a direct statement to this effect would help to clarify the scope of the study for the reader. |
| 74, 90, 103, 113, 117, 118, 160, 168, 276, 288, 316 | Nuckols *et al* vs. Nuckols *et al*. throughout – make sure that each reference is formatted consistently with the others. |
| 92 | Nuckol *et al*.’s – formatting of spacing and apostrophe to be revised |
| 88, 98 | In line 113, the authors state that they used the same 13 guidelines as in the Nuckols paper – is there a rationale for why additional new/updated guidelines were not included in this study beyond 2013 (i.e., the guidelines included in the Nuckols 2014 paper)? How was this decided and why? |
| 88-90 | These lines are written to sound like a coincidence that the chosen guidelines were also those that Nuckols analyzed, whereas it is the case that the Nuckols paper was the method of collection of the sample population to the exclusion of all others. The authors’ decision to study the same guidelines as in Nuckols and Nuckols as a tool of inclusion should be clearly stated and then justified (as in lines 90-91. |
| 174 | What is the reason for including only one Canadian guideline? If the rationale is that the authors used Nuckols to identify the sample population, then it must be stated as such; otherwise, it reads repeatedly as though the authors chose and imposed their own exclusionary criteria on the guidelines to come to the same sample as Nuckols provided. (i.e., “The authors identified the population of guidelines by consulting Nuckols’ sample of guidelines. Nuckols included one Canadian and 12 American guidelines, so these were included in our analysis…” |
| 174 | “…appraisal [no comma] (Table 1).” |
| 193 | “…these five” – does this refer to those guidelines referred to in line 191? Perhaps reorganize paragraph’s sentences to clarify. |
| 209 | The authors don’t refer specifically to the content of guidelines, with the exception of addictions. It would be interesting and indeed useful to see the treatment categories of the 13 guidelines. Were the six guidelines addictions guidelines? Either way, clarify why addictions is stated here and for no other analysis of guidelines? Furthermore, what is the significance of members having expertise in addiction? Would this significance of members’ expertise apply respectively to the other guidelines? |
| 224 | Does this sentence intend that the inclusion of patient representatives somehow neutralize the conflicts or was it found that there were no COI (i.e., that the patient representatives did not have financial ties to industry) or that their COI were less important than others’, so could be excluded? Did the guidelines state that the patient representatives were independent from industry? Did the guideline identify their patient groups? |
| 231 | Rephrase, i.e., “This study likely provides a conservative quantification of potential for bias…” |
| 249 | “Significant”: This study does not include statistical analysis, so perhaps remove the word “significant” and replace with more accurate word. |
| 249 | “Influence”: This study did not quantify the bias, just the elements/characteristics most likely to increase the risk for bias - clarify |
| 257 | “some” – quantify |
| 260 | Add in: “…**risk for** pervasive influence…” |
| 277 | Replace “another study” with “Eady et al. (60),…” |
| 291 | Spacing: AGREEII |
| 291 | Add: “The AGREE II **tool gives**…” |
| 291 | Remove “And” |
| 292 | Add: “in **a** minor role **a** full (word choice, rephrase “full”)… |
| 292 | Change from “marks” to “score” |
| 293 | Add “supports **having** a methodologist…” |
| 293 | Change: “…major role**, since**…” |
| 299 | Individuals (remove comma) |
| 307 | Remove “however”, begin sentence with “A…” |
| 331 | Word choice |
| 336 | Change dashes to commas |
