# Supplementary material for: Drivers of the opioid crisis: An appraisal of financial conflicts of interest in clinical practice guideline panels at the peak of opioid prescribing
Source: PLoS One. 2020 Jan 24;15(1):e0227045. doi: 10.1371/journal.pone.0227045 (PMC6980493; doi:10.1371/journal.pone.0227045)
Supplement: Attachment — Submitted filename: Reponses to reviewers dec 1 2019.docx [file pone.0227045.s004.docx]

**Reponses to reviewers Nov 29 2019**

Dear reviewers,

Thank you for reviewing our draft manuscript. The suggested revisions have strengthened the paper.

We were unable to get permission from Annals of Internal medicine to reproduce and publish the Nuckols data in our table under a CC BY licence. Therefore, we have removed the information from Table 4. and cited it in the text in the results section instead. The changes to the text are highlighted in yellow.

In addition to the comments from Reviewer 2 I have some additional points. Reviewer 2 used the line numbers from the marked up revision rather than the clean copy and so to avoid confusion

I’ll also use the line number in the marked up copy.

1. Line 62: Also include the generic name as well as the brand name.

We have modified this to include (oxycodone)

2. Lines 214-216: The comment by reviewer 2 about these lines appears to be due to problems reading the marked up text and do not have to be addressed.
3. By using the systematic review by Nuckols the authors are accepting that this review uncovered all of the English language guidelines that existed at the time, but the authors should acknowledge that Nuckols may have missed English language guidelines.

We have added this to the limitations section.

“It is also possible that Nuckols *et al.* missed English language guidelines (despite an appropriate search strategy).”

Reviewer #3: Major comments
1. The authors have largely addressed my concerns. However, two important issues remain.

2. Lines 215-216: “Therefore, in discussion, we decided we would use expertise in have with chronic pain or addiction s as a proxy for a balanced committee expertise.”

The explanation provided still does not make sense to me.

We have deferred to editor (see above) for this comment

3. Line 243-244: “We extracted the score for overall quality rating. This score is not an average score. It is a global score reported by reviewers ’after rating all the domains of quality. It is based on this information but is not calculated from those scores”.

This verbiage remains unclear – it appears you are using the overall qualitative assessment suggested for AGREE-II, which is not a “score” as the latte implies a quantitative value, does it not?

The AGREE-II asks the reviewer to assign an overall quality rating as a number between 1 and 7. The user manual on page 8 refers to assigning a global rating and instructs reviewer on when to give a score of 1 or 7, or in between.

We have added throughout the document that the scores are the scores for an overall quality rating.

Minor comments
1. Lines 97 to 108 appear to be primarily methods and not introduction. In addition they appear to be redundant with information on page 7. Consider revising and deleting some of the text in the introduction.

We have removed these lines from the introduction and modified it.

“Our study objective was to appraise the risk of bias from financial conflicts of interest with the pharmaceutical industry in the guidelines included in the 2014 systematic review and critical appraisal by Nuckols *et al.* (25).”

2. Lines 177-178 – some text appears to be missing.

We have fixed the sentence.

“The goal of the group was to inform patients and those that develop, publish and use guidelines.”
